# Pharmacogenomics of Prostaglandin and Leukotriene Receptors

**DOI:** 10.3389/fphar.2016.00316

**Published:** 2016-09-21

**Authors:** José A. Cornejo-García, James R. Perkins, Raquel Jurado-Escobar, Elena García-Martín, José A. Agúndez, Enrique Viguera, Natalia Pérez-Sánchez, Natalia Blanca-López

**Affiliations:** ^1^Research Laboratory, International Business Information Management Association (IBIMA)-Regional University Hospital of Malaga, University of Málaga (UMA)Malaga, Spain; ^2^Allergy Unit, International Business Information Management Association (IBIMA)-Regional University Hospital of Malaga, University of Málaga (UMA)Malaga, Spain; ^3^Department of Pharmacology, University of ExtremaduraCaceres, Spain; ^4^Genetics Unit, Department of Cell Biology, Genetics and Physiology, Faculty of Sciences, University of MálagaMalaga, Spain; ^5^Allergy Service, Infanta Leonor University HospitalMadrid, Spain

**Keywords:** prostaglandins, leukotrienes, eicosanoid receptors polymorphisms, inflammation, NSAID-hypersensitivity

## Abstract

Individual genetic background together with environmental effects are thought to be behind many human complex diseases. A number of genetic variants, mainly single nucleotide polymorphisms (SNPs), have been shown to be associated with various pathological and inflammatory conditions, representing potential therapeutic targets. Prostaglandins (PTGs) and leukotrienes (LTs) are eicosanoids derived from arachidonic acid and related polyunsaturated fatty acids that participate in both normal homeostasis and inflammatory conditions. These bioactive lipid mediators are synthesized through two major multistep enzymatic pathways: PTGs by cyclooxygenase and LTs by 5-lipoxygenase. The main physiological effects of PTGs include vasodilation and vascular leakage (PTGE2); mast cell maturation, eosinophil recruitment, and allergic responses (PTGD2); vascular and respiratory smooth muscle contraction (PTGF2), and inhibition of platelet aggregation (PTGI2). LTB4 is mainly involved in neutrophil recruitment, vascular leakage, and epithelial barrier function, whereas cysteinyl LTs (CysLTs) (LTC4, LTD4, and LTE4) induce bronchoconstriction and neutrophil extravasation, and also participate in vascular leakage. PTGs and LTs exert their biological functions by binding to cognate receptors, which belong to the seven transmembrane, G protein-coupled receptor superfamily. SNPs in genes encoding these receptors may influence their functionality and have a role in disease susceptibility and drug treatment response. In this review we summarize SNPs in PTGs and LTs receptors and their relevance in human diseases. We also provide information on gene expression. Finally, we speculate on future directions for this topic.

## Introduction

Arachidonic acid is released from cell membrane phospholipids by phospholipase A2 in response to different stimuli, and then oxidized through cyclooxygenase (COX)-1 or 5-lipoxygenase (5LO) to produce prostaglandins (PTGs) and leukotrienes (LTs), respectively (Capra et al., 2015). Although these bioactive lipid mediators, collectively named eicosanoids, are associated with homeostasis they also play a key role in inflammation.

The COX-1 pathway firstly leads to the formation of prostanoids with the PTGH2 endoperoxide intermediate, that is metabolized to PTGD2, PTGE2, PTGF2a, PTGI2, and thromboxane (TX)A2 by specific synthases. The main physiological effects of PTGs include vasodilation and vascular leakage (PTGE2); mast cell maturation, eosinophil recruitment and allergic responses (PTGD2); vascular and respiratory smooth muscle contraction (PTGF2), and inhibition of platelet aggregation (PTGI2) (Claar et al., 2015).

LTs can be grouped into the chemoattractant LTB4 and the cysteinyl LTs (CysLTs): LTC4, LTD4, and LTE4. LTB4 is mainly involved in neutrophil recruitment, vascular leakage, and epithelial barrier function, whereas CysLTs induce bronchoconstriction and neutrophil extravasation, and also participate in vascular leakage (Singh et al., 2013).

PTGs and LTs exert their biological functions by binding to cognate receptors belonging to the seven transmembrane G protein-coupled receptor superfamily (GPCRs): PTGFR and PTGIR for PTGF and PTGI2, respectively; PTGDR and CRTH2 (chemoattractant receptor-homologous molecule expressed on Th2 cells) for PTGD; PTGER1 to PTGER4 for PTGE; and TXA2R for TXA2 (Thompson et al., 2014). LTB receptors are BLTR1 and BLTR2, and CysLTR1 and CysLTR2 for CysLTs.

In addition to the environment, human complex diseases are also affected by individual factors including genetic background. In fact, a number of genetic variants, mainly single nucleotide polymorphisms (SNPs), have been consistently associated with different pathological and inflammatory conditions, representing potential targets for therapy. SNPs in genes encoding PTG and LT receptors may influence their functionality and have a role in disease susceptibility and treatment response. In this review we summarize current knowledge regarding SNPs in PTGs and LTs receptors and their association with human inflammatory conditions (Table 1), with a special focus on hypersensitivity to non-steroidal anti-inflammatory drugs (NSAIDs), the most common type of drug hypersensitivity.

**Table 1:** **Main prostaglandin and leukotriene polymorphisms associated with different human inflammatory conditions**.

**Gene**	**Polymorphism**	**Condition**	**Population**	**References**
*Prostaglandin I2 receptor (PTGIR)*	A984C	Cerebral infarction	Japanese	Shimizu et al., 2013
	V53V, S328S	Deep vein thrombosis	Italian	Patrignani et al., 2008
	1915T>C	NERD	Korean	Thompson et al., 2007
*Prostaglandin D2 receptor (PTGDR)*	−441C>T, −549T>C, and −197T>C haplotypes	Asthma	American	Oguma et al., 2004
	−613C>T	Allergy to pollen and mites	Spanish	Isidoro-García et al., 2011
	−197T>C	Allergic asthma	Spanish	Isidoro-García et al., 2011
	−613C>T, −549T>C, −441C>T, and −197T>C diplotype	Nasal polyposis with and without asthma, and aspirin triad	Spanish	Benito Pescador et al., 2012
	−731A>G, 6651C>T	Asthma, atopic asthma, and bronchial hyperreactivity	British and Danish	Zhu et al., 2007
	rs8004654	NIUA	Spanish	Cornejo-Garcia et al., 2012
*Prostaglandin D2 receptor 2 (CRTH2)*	1544G>C, 1651A>G	Asthma and IL13 levels	Chinese	Wang et al., 2009
	1538A>G	Food allergy sensitization	German	Cameron et al., 2009
	−466T>C	NERD	Korean, and Japanese	Palikhe et al., 2010; Kohyama et al., 2012
	−466T>C	Antihistamine requirements in chronic urticaria	Korean	Palikhe et al., 2009
*Prostaglandin E receptor 1 (PTGER1)*	1544G>C, 1651A>G	Asthma	African American, Chinese	Huang et al., 2004
	rs3810253, rs3810255	NERD	Spanish	Ayuso et al., 2015
*Prostaglandin E receptor 2 (PTGER2)*	rs17197	Essential hypertension	Japanese	Sato et al., 2007
	−616C>G, −166G>A	NERD	Korean	Kim et al., 2007b
	rs1254598	NERD	Spanish	Ayuso et al., 2015
*Prostaglandin E receptor 3 (PTGER3)*	rs977214	Protective effect in preterm birth	Norwegian	Ryckman et al., 2010
	1388T>C	Increased asthma risk	Korean	Park et al., 2007
*Prostaglandin E receptor 4 (PTGER4)*	rs7720838	Crohn disease	American and German, German	Glas et al., 2012; Prager et al., 2014
	rs4434423	Primary graft dysfunction	Multicentered cohort including different ethnicities	Diamond et al., 2014
	rs10440635	Ankylosing spondylitis	Han Chinese	Chai et al., 2013
	−1254G>A	NERD	Korean	Kim et al., 2007b
	rs13186505, rs4133101	NIUA	Spanish	Cornejo-Garcia et al., 2012
	rs4495224	NERD	Spanish	Ayuso et al., 2015
	−1254G>A	NECD	Korean	Palikhe et al., 2012
*Thromboxane A2 receptor (TBXA2R)*	rs768963	Cerebral infarction	Han Chinese	Shao et al., 2015
	rs768963	Cerebral infarction, large artery atherosclerosis, and small artery occlusion subtype.	Chinese	Zhao et al., 2013
	795T>C	NERD	Korean, Japanese	Kim et al., 2007b; Kohyama et al., 2012
*Cysteinyl leukotriene receptor 1 (CYSLTR1)*	G300S	Atopy and asthma	Tristan da Cunha	Thompson et al., 2007
	634C>T	NECD	Korean	Kim et al., 2007a
	rs320995	Asthma	Spanish	Arriba-Mendez et al., 2006
	927T>C	Atopy severity	British	Hao et al., 2006
	rs320995	NIUA	Spanish	Cornejo-Garcia et al., 2012
	−634C>T, −475A>C, −336A>G	NERD	Korean	Kim et al., 2006
	−634C>T	NERD	Korean	Kim et al., 2007c
*Cysteinyl leukotriene receptor 2 (CYSLTR2)*	M201V	Atopy	Tristan da Cunha	Thompson et al., 2003
	601A>G	Asthma	Danish and American	Pillai et al., 2004
	−1220A>C	Asthma	Japanese	Fukai et al., 2004
	rs320995	NERD	Spanish	Ayuso et al., 2015
	−819T>G, 2078C>T, 2534A>G	NERD	Korean	Park et al., 2005

NECD, non-steroidal anti-inflammatory drugs-exacerbated cutaneous disease; NERD, non-steroidal anti-inflammatory drugs-exacerbated respiratory disease; NIUA; non-steroidal anti-inflammatory drugs-induced urticaria/angioedema.

## PTG, TBXA2, and LT receptor polymorphisms in inflammatory conditions

### *PTGFR, PTGIR, PTGDR*, and *CRTH2*

Current data concerning *PTGFR* genetic variation supports its relationship with variability in treatment response to latanoprost, a commonly prescribed antiglaucomatous drug, although the mechanisms underlying this association are unknown (Gao et al., 2015; Ussa et al., 2015).

PTGI2 (prostacyclin) plays a key role in the cardiovascular system through the specific inhibition of platelet aggregation and its vasodilatory effects on smooth muscle (Dorris and Peebles, 2012). The polymorphism A984C in *PTGIR* has been associated with platelet aggregation and may play a role in cerebral infarction (Shimizu et al., 2013). Two synonymous variants in this gene (V53V and S328S) have also been associated with enhanced platelet activation in patients with deep vein thrombosis (Patrignani et al., 2008).

An important genomic region on chromosome 14 that includes *PTGDR* has been linked to asthma and atopy (1997; Mansur et al., 1999; Hakonarson et al., 2002). There are conflicting results concerning the association of promoter variants influencing the transcription of this gene with these phenotypes. For example, some studies have failed to associate two variants in *PTGDR* (−441C>T and −197TC>T) with asthma using family-based or case-control analyses in Latinos and African American asthmatics (Tsai et al., 2006). In addition, particular *PTGDR* haplotypes could not be associated with Chinese children with asthma and atopy (Leung et al., 2009), or with two Australian populations of asthmatics (Jamrozik et al., 2011), with similar data having been previously reported in a distinct Chinese population (Li et al., 2007). However, −441C>T, −197TC>T, −549T>C, and various haplotypes have been associated with asthma in two ethnically different populations (Oguma et al., 2004). Moreover, the −197T >C and −613C >T polymorphisms have been significantly associated with allergic asthma, and allergy to pollen and mites, respectively (Isidoro-García et al., 2011). The same authors also found that the *PTGDR* diplotype CCCT/CCCC (−613CC, −549CC, −441CC, and −197TC), with a potential influence on gene transcription, was more frequent in patients with nasal polyposis, with or without asthma, and in patients suffering from the aspirin triad (Benito Pescador et al., 2012). In addition to these SNPs in *PTGDR*, the promoter polymorphism −731A>G and the intronic variant 6651C>T were found to be associated with asthma, atopic asthma, and bronchial hyperreactivity in families from UK, with similar results for 6651C>T in a Danish study (Zhu et al., 2007). The intronic variants rs17831675 and rs17831682 in *PTGDR* have also been associated with asthma susceptibility (Ungvári et al., 2012). These variants, as well as rs17125273, also appear to have some relevance with respect to IgE levels in asthmatics (Ungvári et al., 2012).

Different studies have shown certain polymorphisms in *CRTH2* (rs11571288, rs545659, and rs634681) to be associated with asthma and allergic sensitization in different populations (Huang et al., 2004; Cameron et al., 2009; Wang et al., 2009). A functional study showed that in addition to being linked with asthma, the 1544G–1651G haplotype in the *CRTH2* 3′-UTR increased mRNA stability, supporting its role as a strong candidate gene for asthma (Huang et al., 2004). In another study the rs533116 variant was associated with a higher proportion of CRTh2 cells during Th2 differentiation as well as increased IL-4 and IL-13 expression levels after agonist stimulation (Campos Alberto et al., 2012). The minor allele of *CRTH2* 1431G>C has been also associated with levels of specific IgE to food allergens in a population of German children (Cameron et al., 2009).

### PTGE receptors

Considering the great variety of biological functions carried out by PTGE it is not surprising that SNPs in its receptors appear to have a role in different pathologies. For example, the rs17197 polymorphism in the 3′-UTR of *PTGER2* has been associated with essential hypertension in men (Sato et al., 2007), and the intronic rs2268062 variant in *PTGER3* with hypertension in a separate study (Sõber et al., 2009). The latter may be explained by the role of this gene in neurotransmitter release modulation in central and peripheral neurons, and in the inhibition of sodium and water reabsorption in kidney (Breyer and Breyer, 2000). A protective effect in preterm birth has been suggested for the minor allele of rs977214 in *PTGER3* (Ryckman et al., 2010).

Concerning asthma, a Bayesian analysis found an association for several SNPs in *PTGER2* (rs12587410, rs17197, rs1254600, rs708498) (Ungvári et al., 2012). A Korean study showed the promoter variant rs207579 (−616C>G) in *PTGER2* to be associated with increased asthma risk whereas two variants in the 3′-UTR of *PTGER3* (rs959 and rs34745168) were associated with a diminished risk (Park et al., 2007).

Environmental and genetic determinants affecting *PTGER4* expression might be thought to be crucial in a plethora of diseases, giving the central role of its ligand, PTGE2, in immunomodulation and control of inflammation (Soontrapa et al., 2011; Tang et al., 2012; Konya et al., 2013). For example, it has been shown for Crohn's disease that disease-associated alleles in a gene-desert region in 5p13.1 correlate with expression levels of *PTGER4*, the closest gene to this region (Libioulle et al., 2007). Through a meta-analysis of genome wide association studies and replication studies, rs11742570 in *PTGER4* was recently found to be associated with inflammatory bowel disease (Jostins et al., 2012).

Two polymorphisms in *PTGER4* (rs4495224 and rs7720838) have been associated with susceptibility to Crohn's disease in three independent cohorts (Glas et al., 2012). Using *in silico* analysis the authors predicted these SNPs to be crucial components of binding sites for the transcription factors NF-κB and XBP1 with higher binding scores for carriers of the risk alleles, which could explain how these SNPs contribute to increased *PTGER4* expression (Glas et al., 2012). Notably, the association of *PTGER4* rs7720838 with Crohn's disease has recently been replicated in other populations (Prager et al., 2014).

Although the molecular link between genetic variation and altered regulatory T cell function in primary graft dysfunction has not been uncovered, a recent study showed the major allele of the rs4434423 variant in *PTGER4* to be associated with their differential suppressive functions in this pathology (Diamond et al., 2014).

Finally, another SNP in *PTGER4* (rs10440635) has been associated with ankylosing spondylitis severity in a Han Chinese population (Chai et al., 2013).

###  *TXA2R*

*TXA2R* plays a central role in atherosclerosis and thrombosis (Capra et al., 2014). It has been recently demonstrated that the promoter polymorphism rs768963 is more frequent among Han Chinese who have suffered from cerebral infarction (Shao et al., 2015). Cells containing two exonic variants (C795T and T924C) showed an increased ligand binding-induced intracellular calcium influx and fibrinogen-integrin conjugation, suggesting that *TXA2R* SNPs may influence platelet function and the risk of developing cerebral ischemia (Shao et al., 2015). In another study the C allele of rs768963 was associated with large artery atherosclerosis and small artery occlusion subtypes (Zhao et al., 2013).

The variants V80E and A160T in *TBXA2R* showed opposing roles on platelet activity, inhibiting and promoting platelet activation respectively (Gleim et al., 2013). The *TBXA2R* 924TT genotype has been associated with residual platelet activity after off-pump artery bypass grafting (Wang et al., 2013), and with resistance to aspirin (Gao et al., 2011). This genotype also showed less effectiveness in platelet aggregation (Fujiwara et al., 2007). Homozygosity for the minor allele of the C795T, C924T or the G1686A *TBXA2R* SNPs was associated with a decreased expression of CD62P in agonist stimulated platelets (Fontana et al., 2006). Finally, the *TBXA2R* SNP rs13306046 was associated with decreased blood pressure (Nossent et al., 2011).

A recent meta-analysis has shown an association between the *TBXA2R* 924C>T polymorphism and asthma (Pan et al., 2016). Asthmatic children with combinations of the *TBXA2R* 795T>C and 924T>C risk alleles had higher total IgE levels, total eosinophil counts, and lower FEV1 than those carrying the common alleles (Kim et al., 2008). The minor allele frequency of *TBXA2R* 795T>C was significantly higher in NSAIDs-exacerbated respiratory disease (NERD) patients compared with aspirin-tolerant asthmatics and homozygous patients for the C allele had a greater percent fall of FEV1 compared with carriers of the CT or TT genotypes (Kim et al., 2005). A significant association was also observed between the *TBXA2R* T924C polymorphism and FEV1 in children with atopic asthma (Leung et al., 2002; Hong et al., 2005).

Two haplotypes involving four intronic SNPs (rs2238631, rs2238632, rs2238633, and rs2238634) influenced transcriptional activity and were associated with asthma-related phenotypes (Takeuchi et al., 2013). These SNPs are in linkage disequilibrium with the exonic SNPs rs11318632 and rs4523. The first one was associated with atopic asthma (Shin et al., 2003), and the latter with adult asthma (Unoki et al., 2000) and atopic asthma in children (Leung et al., 2002). A suggestive association between rs8113232 in *TBXA2R* and rhinitis in asthmatic children has been also reported (Kavalar et al., 2012). Finally, the *TBXA2R* 4684T allele is associated with lower *TBXA2R* expression, which may contribute to the development of NSAIDs-exacerbated cutaneous disease (NECD) (Palikhe et al., 2011).

### *LTB4R1* and *LTB4R2*

Gene structure analysis of lung samples has revealed that both *LTB4R1* and *LTB4R2* show splice variation in the 5′-UTR and multiple promoter regions, although they did not find susceptibility markers for asthma development (Tulah et al., 2012). A previous study failed to associate polymorphisms spanning the *LTBR* locus with baseline lung function in smokers (Tulah et al., 2011).

Increased cytotoxicity and migration of NK cells appears to be linked to LTB4 (Wang et al., 2015). However, there is a differential role for its two receptors. Using a selective receptor antagonist, LTB4R1 was shown to be involved in both LTB4-induced migration and cytotoxicity, whereas LTB4R2 was involved exclusively in NK cell migration, but only in response to higher concentrations of LTB4. The expression of these two receptors increased after activation of NK cells with IL-2 and IL-15, and these changes in expression were reflected in enhanced NK cell responses to LTB4 (Wang et al., 2015).

### *CYSLTR1* and *CYSLTR2*

The functional variant G300S in *CYSLTR1* was associated with atopy in the Tristan da Cunha population (Thompson et al., 2007) as the M201V SNP in *CYSLTR2* was previously (Thompson et al., 2003). The G300S association has been replicated in another study showing that LTD4-induced phosphorylation of *Erk* is higher in transfected cells (Yaddaden et al., 2016).

Significant differences in genotype frequencies of the *CYSLTR1* promoter polymorphism −634C>T have been associated with clinical requirement for leukotriene receptor antagonists (Kim et al., 2007a). In addition, patients with the variant genotype showed higher expression levels of *CYSLTR1* than those with the common genotype (Kim et al., 2007a).

The rs320995 variant in *CYSLTR1* has been associated with asthma in two independent studies in Spain (Arriba-Mendez et al., 2006; Sanz et al., 2006). Although a UK study involving 341 families could not find this association, it was linked to atopy severity in females (Hao et al., 2006).

A significant association between the coding polymorphism 601A>G in *CYSLTR2* and asthma was observed in a family-based study of asthmatics from Denmark and USA, with the G allele appearing to be less frequently transmitted to asthmatics (Pillai et al., 2004). Using a calcium mobilization assay the authors demonstrated that carriers of the G allele, LTD4 was approximately five-fold less potent compared to wild type, suggesting a potential mechanism for resistance to asthma (Pillai et al., 2004).

A transmission disequilibrium test of 137 Japanese asthmatic families showed that the −1220A>C SNP in *CYSLTR2* was associated with asthma development and with atopic asthma in Japan (Fukai et al., 2004).

The mRNA and protein expression of *CYSLT1R* and *CYSLT2R* was found to be significantly increased in polyp tissues from eosinophilic chronic rhinosinusitis patients, and this increase was shown to be related to LTC4 and LTD4 levels (Wu et al., 2016). *CYSLTR2* is expressed in the lungs of asthmatics and its activation appears to contribute to antigen-induced bronchoconstriction in certain asthma populations (Sekioka et al., 2015).

## PTG, TBXA2, and LT receptor polymorphisms in NSAID hypersensitivity

NSAIDs, commonly used in the treatment of pain and some inflammatory diseases, are the main triggers of drug hypersensitivity reactions (Conaghan, 2012). The most frequent type of reaction caused by these drugs is cross-intolerance, in which COX-1 inhibition leads to CysLTs release, resulting in clinical symptoms in susceptible individuals (Doña et al., 2011, 2012, 2014). Three main phenotypes of cross-intolerance are currently recognized: NERD (previously known as aspirin-induced asthma and ASA triad), in patients with rhinitis and/or asthma with/without nasal polyposis; NECD (previously known as aspirin-intolerant chronic urticaria), in patients with underlying chronic idiopathic urticaria; and NSAIDs-induced urticaria/angioedema (NIUA) in otherwise healthy individuals (Kowalski et al., 2013).

Recent years have witnessed increased interest in the potential role of SNPs in these pathologies. We will now focus on those variants related to PTG and LT receptors.

### *PTGFR, PTGIR, PTGDR*, and *CRTH2*

Most genetic association studies have not further investigated the functional effects of associated variants, for example, the 1915T>C variant in *PTGIR* that has been associated with NERD in Korea, however the mechanisms by which it leads to the pathology are unclear (Kim et al., 2007b).

Although the *PTGDR* −549T>C (rs8004654) SNP could not been shown to be associated with NERD in a Korean population (Kim et al., 2007b), we recently found an association between NIUA, the most common type of drug hypersensitivity reactions, and this polymorphism in two independent Spanish populations (Cornejo-Garcia et al., 2012). In terms of potential mechanism, it has been shown that the C allele (risk for NIUA in our study) increases GATA-2/GATA-3 transcription factor binding, modifying promoter activity and *PTGDR* gene expression (Oguma et al., 2004). Interestingly, given that an association could not be found in the Korean study mentioned above, this variant has also been associated with NERD in Spain (Ayuso et al., 2015).

In patients with NERD the *CRTH2* −466T>C polymorphism increases both serum and cellular eotaxin-2 production, and may lead to eosinophilic infiltration in these patients (Palikhe et al., 2010). Another study also showed that the frequency of the homozygous genotype for the less frequent allele of *CRTH2* −466T>C was higher in NERD patients compared to aspirin-tolerant asthmatics (Kohyama et al., 2012). Interestingly, homozygous TT chronic urticaria patients required higher doses of antihistamines (Palikhe et al., 2009).

### PTGE receptors

Two promoter variants (rs3810253 and rs3810255) in *PTGER1* have recently been found to be associated with NERD, although their functional consequences need to be elucidated (Ayuso et al., 2015).

We have found that the rs1254598 polymorphism in *PTGER2* was marginally associated with NERD patients when compared with NSAID-tolerant asthmatics (Ayuso et al., 2015). In addition, two promoter variants in *PTGER2* (−616C>G and −166G>A) have been associated with NERD in a Korean population (Kim et al., 2007b).

It has been shown that in patients with NECD the GG genotype at −1254G>A in *PTGER4* is more frequent than in healthy individuals (Palikhe et al., 2012). In addition, the G allele leads to lower *in vitro* promoter activity with decreased *PTGER4* expression (Palikhe et al., 2012). This promoter polymorphism (−1254A>G) has also been reported to be associated with NERD in Koreans (Kim et al., 2007b). However, recent research failed to demonstrate an association between NIUA and two promoter variants in *PTGER4* (rs13186505 and rs4133101) in a Spanish population (Cornejo-Garcia et al., 2012). In addition, no statistically significant association was found for another variant in *PTGER4* (rs4495224) in NERD patients (Ayuso et al., 2015).

###  *TXA2R*

The frequencies of the CC/CT genotype of *TBXA2R* 795T>C were higher than those of the TT genotype in NERD patients compared to ASA-tolerant asthmatics (Kohyama et al., 2012). *TBXA2R* −4684C>T and 795T>C variants have also been associated with NERD (Kim et al., 2007b).

### *CYSLTR1* and *CYSLTR2*

Male NERD patients showed higher frequencies of the minor alleles of −634C>T, −475A>C, −336A>G polymorphisms in *CYSLTR1* than male controls (Kim et al., 2006). In addition, the haplotype [T-C-G] was associated with increased disease risk for NERD and also affected gene expression, measured by significantly enhanced luciferase activity (Kim et al., 2006). The −634C>T SNP was also most frequent in NERD compared to NECD patients and the variant genotype showed significantly higher promoter activity than the common genotype (Kim et al., 2007c).

The *CYSLTR1* variant rs320995 was associated with NIUA in two independent Spanish populations (Cornejo-Garcia et al., 2012). It has also been associated with NERD in Spain (Yaddaden et al., 2016), but not with NERD in Korea (Choi et al., 2004).

Finally, NERD patients carrying the minor alleles for 819T>G, 2078C>T or 2534A>G in *CYLSTR2* exhibited a more pronounced fall in FEV1 after aspirin provocation than those who carried the common allele (Park et al., 2005).

## Future directions

Important international efforts such as HapMap and the 1000 Genomes projects, as well as advances in *in silico* analysis have led to substantial advances in our knowledge of the human genome and its relationship with disease. This will almost certainly increase in the near future through the adoption of next-generation sequencing approaches for the discovery of associated variants, and the integration of the different omics technologies, such as transcriptomics, metabolomics, and lipidomics, which will help us to uncover the underlying mechanisms behind complex human diseases.

Focusing on GPCRs, a number of public databases integrating genome data and *in silico* predictions are currently available and have been recently reviewed (Thompson et al., 2014). Four recommended computational tools for predicting the functional effects of SNPs on protein structure and function are PolyPhen (http://genetics.bwh.harvard.edu/pph2), SIFT (http://sift.jcvi.org), SNAP (https://www.broadinstitute.org/mpg/snap), and Mutation Taster (http://www.mutationtaster.org).

Mediators from the arachidonic acid pathway play a key role in inflammation as well as in the pathogenesis of a number of diseases. The lack of agreement in genetic association studies is likely related to population differences, including ethnic genetic background, sample size, and differences in phenotype definitions. In addition, most genetic studies have not addressed rare variants that are thought to be involved in complex diseases together with environmental factors. In spite of these limitations, as technologies improve and international networks and collaborations continue to flourish, we expect our knowledge on the genetic basis of inflammation and other conditions to become more refined, potentially leading to the discovery of markers and treatment options for a range of diseases.

## Author contributions

JC, JP, RJ, NS, and NB revised the literature available on this topic and wrote the first manuscript draft. JC, EG, JA, and EV revised the manuscript. JC and JP are responsible of the final English version.

## Funding

JC receives funding from the Miguel Servet Program (Ref CP14/00034); JP receives funding the Sara Borrell Program (Ref CD14/00242), both from the Carlos III National Health Institute, Spanish Ministry of Economy and Competitiveness (grants co-funded by the European Social Fund, ESF). The present study was supported by grants from the Carlos III National Health Institute, Spanish Ministry of Economy and Competitiveness (grants cofunded by the European Regional Development Fund, ERDF): RD12/0013/0001 (Red de Investigación de Reacciones Adversas a Alérgenos y Fármacos, RIRAAF Network), FIS PI12/02247, FIS PI13/02598, FISPI15/00726; the Andalusian Public Health Service (PI-0279-2012); and Junta de Extremadura (Grant GR15026, cofounded by European Regional Development Fund, ERDF).

### Conflict of interest statement

The authors declare that the research was conducted in the absence of any commercial or financial relationships that could be construed as a potential conflict of interest.
